# Personalized prevention in oncology: integrating the current approaches for the benefit of population health

**DOI:** 10.1093/eurpub/ckac163

**Published:** 2022-11-09

**Authors:** Stefania Boccia, Walter Ricciardi

**Affiliations:** Section of Hygiene, Department of Life Sciences and Public Health, Università Cattolica del Sacro Cuore, Rome, Italy; Department of Woman and Child Health and Public Health, Fondazione Policlinico Universitario A. Gemelli IRCCS, Roma, Italy; Section of Hygiene, Department of Life Sciences and Public Health, Università Cattolica del Sacro Cuore, Rome, Italy

Although Europe accounts for a 10th of the world’s population, a quarter of the world’s cancer cases are diagnosed in Europe every year. According to the latest available data, 2.7 million people were diagnosed with cancer in 2020, and another 1.3 million people lost their lives to it.^1^ In terms of Disability Adjusted Life Years and mortality rates, no major changes have been reported in the past 30 years, with neoplasms remaining in both cases in the second position after cardiovascular diseases.^1^ This figure, unfortunately, is expected to increase rapidly due to ageing populations, unhealthy lifestyles, unfavourable health determinants and environmental and working conditions unless urgent measures are taken.

The European Beating Cancer Plan (EBCP) published in February 2021, represents the EU’s renewed commitment to cancer prevention, treatment and care, and it is structured around the four key action areas of understanding, prevention, diagnosis, treatment and quality of life.^2^ The plan recognizes that between a third and a half of cancer cases could be prevented if the current understanding about risk factors was translated into effective public health actions, so that dedicated investments will be provided to implementation research in the prevention pillar. In addition, the implementation plan of EBPC reports the needs for more understanding of novel personalized effective measures for cancer prevention. As such, dedicated funding to: (i) develop new methods and technologies for screening and early detection (e.g. ‘integrated diagnostics’—imaging, tissue, fluid, clinical biomarkers, also using Artificial Intelligence), and (ii) develop early predictors/tests, have been released and will continue until the end of the Horizon Europe program.

The need to innovate the current model of cancer prevention is urgently needed: the latest available data suggest that there is no linear relationship between investments in preventive care as share of expenditure of healthcare, and the healthy life years at birth, e.g. Estonia and Finland that spend in prevention more than the average EU expenditure in healthcare, experience the worst length of healthy life years at birth.^3^ Personalized prevention brings the promise to increase the effectiveness and efficiency of preventive interventions, and it revolves around the adoption of targeted actions that combine the biological information (e.g. genetics and other biomarkers, demographics, health condition), with environmental and behavioural characteristics, and socio-economic and cultural context of individuals.^4^ For cancer, risk predictions based on age and sex are useful, as both are strongly associated with risk. However, as knowledge into the causes of disease has improved, additional environmental and biological factors have been identified as associated with disease. In many common cancers, comprehensive risk prediction models (RPMs) that combine a set of factors, can provide a risk estimate, however, the effort and cost necessary to collect the information will also affect decisions on inclusion in risk prediction for use in preventive and clinical care.

If we consider primary prevention in cancer, where unhealthy lifestyles have the larger impact in terms of avoidable morbidity, evidence suggests to evaluate the potential role of polygenic scores (PRS, common inherited genetic variants contributing to disease risk) as a measure of genetic contribution to the risk of developing cancer to be used independently or as part of RPMs to trigger lifestyle changes by targeting personalized educational efforts.^5^ The same holds for secondary prevention: the use of PRS has been demonstrated to have good predictive power for identifying women at the highest and lowest risk of breast cancer and could be used to improve breast screening programs. Risk-stratified screening approaches for breast cancer are being investigated in a number of studies that are expected to report initial or further results in the next few years. Continued effort is needed to gather the appropriate evidence for evaluation and demonstration of the utility of such innovative tools that would support implementation efforts, as premature implementation of PRS in cancer risk estimation approaches could undermine these efforts, and risk loss of confidence in this potentially valuable area of population health improvement.

Large inequalities in health status and life expectancy exist across population groups and within the OECD countries, and these inequalities are linked to many factors, including differences in exposure to health risk factors and in access to health care. The potential for personalized prevention to improve population health comes with the real risk that benefits will not materialize for all people. It is urgent to identify priorities and actions that can help ensure that everyone has the opportunity to reap the health benefits of advances in personalized prevention in cancer, by involving researchers, healthcare workers, patients and citizens and policy makers, and by properly planning translation of research findings.

*Conflicts of interest*: None declared.
